# Genetic Differentiation of Bisexual and Parthenogenetic Populations of Plant Louse *Cacopsylla ledi* (Hemiptera, Psylloidea)

**DOI:** 10.3390/insects16121268

**Published:** 2025-12-13

**Authors:** Nazar A. Shapoval, Seppo Nokkala, Christina Nokkala, Galina N. Shapoval, Eugenia S. Labina, Anna E. Romanovich, Valentina G. Kuznetsova

**Affiliations:** 1 Department of Karyosystematics, Zoological Institute, Russian Academy of Sciences, Universitetskaya emb. 1, 199034 St. Petersburg, Russia; galinakuftina@mail.ru (G.N.S.); labina_e@mail.ru (E.S.L.); 2 Laboratory of Genetics, Department of Biology, University of Turku, FI-20014 Turku, Finland; sepnok@utu.fi (S.N.); chrinok@utu.fi (C.N.); 3 Institute of Biology and Biotechnology, Altai State University, Lenina Ave. 61, 656049 Barnaul, Russia; 4 Resource Center for Development of Molecular and Cellular Technologies, Saint-Petersburg State University, Universitetskaya emb. 7/9, 199034 St. Petersburg, Russia; aromanovich@gmail.com

**Keywords:** bisexual reproduction, DNA barcodes, infection frequency, jumping plant lice, parthenogenesis, PCR-screening, phylogeography, *Wolbachia* infection

## Abstract

The psyllid genus *Cacopsylla* comprises mainly bisexually reproducing species; however, some members of this genus exhibit a unisexual mode of reproduction. Using an integrative approach that combines molecular and cytogenetic methods, as well as *Wolbachia* screening, we conducted a comprehensive study of the Palaearctic species *C. ledi*. We show that this species uses various reproductive strategies (bisexual and parthenogenetic) across its distribution range. Our findings indicate that the bisexual mode of reproduction has emerged at least twice in the evolutionary history of *C. ledi*. Bisexual populations in southern Fennoscandia are of ancestral origin, whereas the bisexual mode of reproduction observed in northern Fennoscandia represents a recent secondary transition from parthenogenesis. We report that in the first case, parthenogenetic and bisexual lineages can be easily distinguished not only cytogenetically but also by DNA barcoding, while in the second case, “bisexual” individuals share DNA barcodes with parthenogenetic ones. A comprehensive *Wolbachia* screening (1140 specimens across the entire distribution range) revealed *Wolbachia* infection in every specimen of *C. ledi*, indicating a significant role of the endosymbiont in the biology and evolution of this species.

## Simple Summary

The vast majority of insects reproduce bisexually, and unisexual species that reproduce via parthenogenesis comprise a negligible fraction of all species. To date, very little is known about the evolutionary potential of parthenogenesis, and there remains little research related to this topic. Parthenogenetic lineages are generally considered short-lived on an evolutionary scale. It is believed that the evolutionary potential of parthenogenetic species is low due to the lack of mechanisms to counteract the effects of accumulation of deleterious mutations, which may explain the observed low incidence of parthenogenetic species among Insecta. Our study fills this gap by analysing over 5000 specimens of *Cacopsylla ledi* using an integrative approach that combines molecular and cytogenetic methods, as well as *Wolbachia* screening. We determined the reproductive mode of psyllids using cytogenetic methods, conducted phylogeographic analysis based on almost 1000 barcoded specimens, and screened specimens for *Wolbachia* infection by amplifying three *Wolbachia*-specific genes. We demonstrate that apomictic triploid parthenogenesis is not necessarily an evolutionary dead end but can lead to the emergence of a new bisexual species and that *Wolbachia* infection can play a significant role in this process.

## 1. Introduction

Psyllids (Hemiptera, Psylloidea), or jumping plant lice, include more than 4000 described species, most of which are monophagous or oligophagous and feed on plant sap [1]. In the superfamily Psylloidea, the genus *Cacopsylla* Ossiannilsson, 1970 is one of the largest and includes about 460 taxa with a predominantly Holarctic distribution [2,3,4]. The vast majority of Psylloidea species reproduce bisexually. However, recent studies have clearly shown that some *Cacopsylla* species can reproduce by thelytoky, a form of parthenogenesis in which females produce all-female progeny from unfertilized eggs [5,6,7,8,9].

Thelytokous parthenogenesis can be apomictic (apomixis) or automictic (automixis). In apomictic parthenogenesis, females undergo modified meiosis, resulting in offspring developing from unfertilized but diploid eggs. This mode of reproduction is strictly clonal, and populations consist exclusively of females. Apomictic parthenogenesis is usually associated with polyploidy [10,11]. In automictic parthenogenesis, females undergo normal meiosis to produce haploid gametes, but diploidy is then restored through various mechanisms [10,12,13].

In the genus *Cacopsylla*, the best-documented parthenogenetic species are *C. borealis* Nokkala et Nokkala, 2019, *C. myrtilli* (Wagner, 1947), and *C. ledi* (Flor, 1861). The recently described *C. borealis* [14] is the only known pentaploid jumping plant louse with apomictic females (males were not found) exhibiting the karyotype of 2n = 5x = 60 + XXXXX. Another well-studied apomictic species is *C. myrtilli*, which has been analysed using both cytological and molecular approaches [5,6,7,15,16,17]. Most populations of *C. myrtilli* consist exclusively of triploid females (2n = 3x = 36 + XXX), although rare diploid females (2n = 24 + XX) and rare males (2n = 24 +X) are occasionally found. These rare diploids are believed to be produced de novo in each generation by apomictic females as a reversion from triploidy. Rare males are non-functional, have aberrant meiosis and are unable to produce viable haploid sperm and, hence, euploid offspring. Molecular studies revealed several populations in Finland and Russia where males exhibit normal meiosis [7,16]. In these populations, diploid females and males possess distinct mitochondrial haplotypes that are not found in parthenogenetic females. This finding highlights the evolutionary potential of parthenogenesis as a driver of speciation: if males produced by parthenogenetic females become functional, then mating with diploid females can result in a new bisexual lineage reproductively isolated from its parthenogenetic ancestor, potentially leading to speciation [14,16].

**Figure 1:**
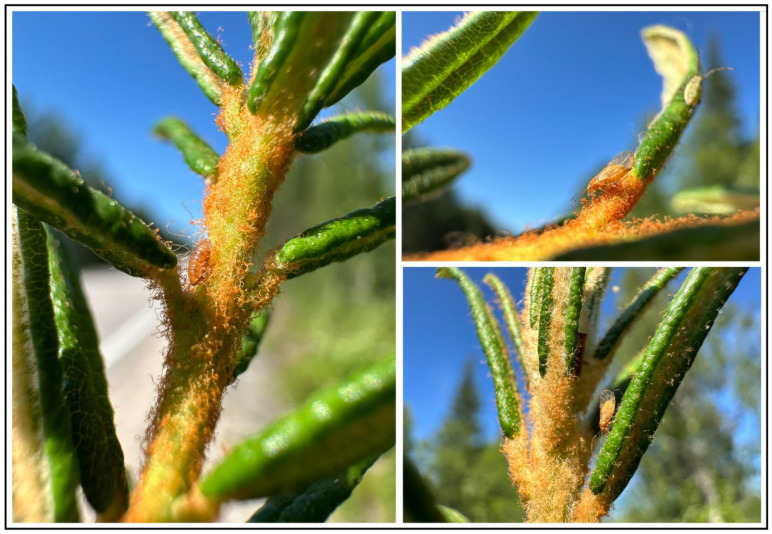
*Cacopsylla ledi* immature individual (**left**) and adult females (**right**) on the host plant (*Ledum palustre*). Russia, Murmansk Oblast, 10 km W of Kandalaksha town, 67.157280° N, 32.150402° E. 20 July 2024. Photos: G.N. Shapoval.

*Cacopsylla ledi* inhabits the boreoalpine zone of the Holarctic [18], develops, lives and feeds on *Ledum palustre* L. 1953 (=*Rhododendron tomentosum* Harmaja, 1990) (Figure 1), and its distribution range matches that of its host plant.

Recent cytological and molecular studies [8,9] have identified two evolutionary lineages of *C. ledi* with different reproductive strategies: the ancestral bisexual lineage and the parthenogenetic lineage. The parthenogenetic lineage is mainly represented by triploid females (2n = 3x = 36 + XXX), although rare males and diploid females have been observed in some populations. Unlike *C. myrtilli*, rare males of *C. ledi* produce normal sperm and are therefore considered functional. The bisexual lineage of *C. ledi* either forms mixed populations with the parthenogenetic lineage, or, which is less common, exists as completely bisexual populations. These findings suggest a complex interaction between the reproductive modes and the evolutionary history of *C. ledi*.

Recent studies have discovered the presence of *Wolbachia* infection in several *Cacopsylla* species, suggesting an essential role of this bacterium in their biology. It is well known, that *Wolbachia* has diverse impacts on its hosts. In particular, *Wolbachia* can skew the sex ratio towards females [19], induce parthenogenesis [20] and significantly affect mtDNA diversity in hosts [21,22,23]. At the intraspecific level, *Wolbachia* can reduce mtDNA polymorphism: when an endosymbiont invades a new host population, the frequency of mitochondrial haplotypes associated with *Wolbachia* increases due to the so-called ‘hitch-hiking’ effect. If infection has occurred recently on an evolutionary scale, mtDNA diversity within the host species may decrease significantly [22,24,25]. At the interspecific level, *Wolbachia* can cause mitochondrial introgression between closely related species, leading to the fixation of alien mtDNA haplotype (-s) in host populations [23,25,26,27,28,29].

The aim of our study was to conduct a large-scale phylogeographic analysis of *C. ledi* with the following objectives: (1) to identify the genetic structure of this species using the mitochondrial molecular marker *COI* and comprehensive taxon sampling, (2) to determine the overall distribution pattern of populations with different reproductive strategies in Fennoscandia, (3) to gain new insights into the evolutionary history, diversification and post-glacial dispersal of this species.

Another objective was to study the incidence and prevalence of *Wolbachia* in *C. ledi*, analyse the impact of endosymbiont on the mtDNA diversity of its host, and determine whether the parthenogenetic and bisexual patterns observed in this species are associated with *Wolbachia* infection.

## 2. Materials and Methods

### 2.1. Taxon Sampling

**Figure 2:**
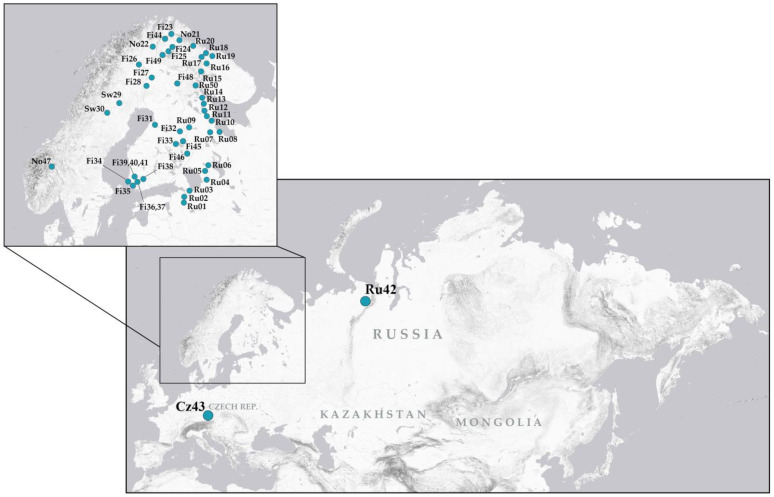
Map showing the sampling localities of *Cacopsylla ledi* across the study area.

Psyllids were collected from various regions of the Palaearctic, with a particular focus on Fennoscandia, during field expeditions conducted between 2007 and 2025. In total, *C. ledi* individuals were sampled from 50 populations: 22 in Russia, 22 in Finland, 3 in Norway, 2 in Sweden, 1 in the Czech Republic (Figure 2, Supplementary Tables S1 and S2). Attempts to locate *C. ledi* in several regions in eastern Russia (Zabaykalsky Krai, Republic of Yakutia, Magadan Oblast), as well as earlier attempts to detect this species in northern Finland, where it is replaced by closely related sympatric species *C. borealis* [9], were unsuccessful despite the abundance of the host plant (*Ledum palustre*). Immediately after capture, individuals were preserved in either 96% ethanol or a 3:1 fixative solution (96% ethanol: glacial acetic acid) and stored for subsequent genetic analyses and cytological studies. Alternatively, for chromosomal and DNA analyses of the same specimens, live individuals were transported to the laboratory and dissected. For each specimen, the abdomen was placed in fixative (chromosomal preparations are made from testes) and the remainder of the body was preserved in 96% ethanol.

### 2.2. Cytological Studies

Cytological analysis was performed following previously described protocols [5]. For specimens collected in ethanol, the method first applied by Nokkala et al. [6] was used, which allows chromosomal and molecular analyses from the same individual. Chromosomal preparations were made, stained, and photographed according to Nokkala et al. [6,7,8,14].

### 2.3. DNA Extraction

Whole specimens or the head and thorax of individual specimens were used for DNA extraction using the DNeasy Blood and Tissue Kit (Qiagen, Inc., Valencia, CA, USA) following the manufacturer’s protocols and the CTAB-based method [30] with modifications [31,32,33]. Samples were processed at the Department of Biology, University of Turku (Finland) and the Department of Karyosystematics, Zoological Institute of the Russian Academy of Sciences (St. Petersburg, Russia).

### 2.4. mtDNA PCR and Sequencing

Fragments of 655 bp and 725 bp in length of the cytochrome c oxidase subunit I (*COI*) gene were amplified using the primer pairs HybCacoCO/HybHCOMod [8] and CACF/CACR [34], respectively. PCR was performed under conditions previously described [7,8,14,34]. PCR products were purified using the QIAquick PCR Purification Kit (Qiagen, Inc., Valencia, CA, USA) or treated with FastAP and ExoI enzymes (Thermofisher, Waltham, MA, USA), and then sent for sequencing to either Macrogen Europe (Amsterdam, The Netherlands) or Evrogen (Moscow, Russia). The obtained *COI* sequences were deposited in GenBank under accession numbers PX465190-PX465303, PX468288-PX468487, PX502613-PX503209, PX600319-PX600326, PX632653-PX632667. For further analysis, we used *COI* alignment that was trimmed to the length of the shortest sequence (655 bp). Maximum parsimony haplotype network was reconstructed to illustrate the relationships among *COI* haplotypes using TCS algorithm [35] implemented in PopART [36]. General sequence information was analysed and neutrality tests were performed using MEGA v.7.0.14 [37] and DnaSP v.6.12.03 [38] software.

### 2.5. Detection of Wolbachia Endosymbionts

To screen *C. ledi* specimens for *Wolbachia* infection, we amplified three *Wolbachia*-specific genes: *16S ribosomal RNA* (*16S rRNA*), *Wolbachia surface protein* (*wsp*), and *filamentation temperature-sensitive protein Z* (*ftsZ*). We used the following primer pairs: W-Specf/W-Specr [39], wsp81F/wsp691R [40], and ftsZ-F/ftsZ-R [41], amplifying ~396 bp fragment of the *16S rRNA* gene, ~549 bp fragment of the *wsp* gene, and ~510 bp fragment of the *ftsZ* gene, respectively (actual fragments sizes may vary depending on the *Wolbachia* strain). PCR conditions and thermal profiles were as previously described [17,33]. To determine *Wolbachia* presence or absence, PCR products were visualised and checked on a 1% agarose gel. For each specimen, PCR was performed twice to minimise the risk of technical error. Samples that yielded products of the expected sizes for the selected genes were considered *Wolbachia*-positive.

To identify the *Wolbachia* allele(s) infecting *C. ledi*, a 549 bp fragment of the *wsp* gene was sequenced for selected specimens. Samples, for which standard Sanger sequencing revealed intra-individual allele polymorphism (in the form of single nucleotide heterogeneities), suggesting co-infection with genetically different *Wolbachia* alleles, were subsequently cloned. The cloning procedure followed the previously described protocols [42,43]. Ten clones of the *wsp* gene per specimen were sequenced. Sequencing of the double-stranded product was performed using an ABI 3500xL analyser (Applied Biosystems, Waltham, MA, USA) at the Research Resource Center for Molecular and Cell Technologies (St. Petersburg State University, St. Petersburg, Russia). The obtained *wsp* sequences were deposited in GenBank under accession numbers PX647420-PX647462.

## 3. Results

### 3.1. Haplotype Diversity

**Figure 3:**
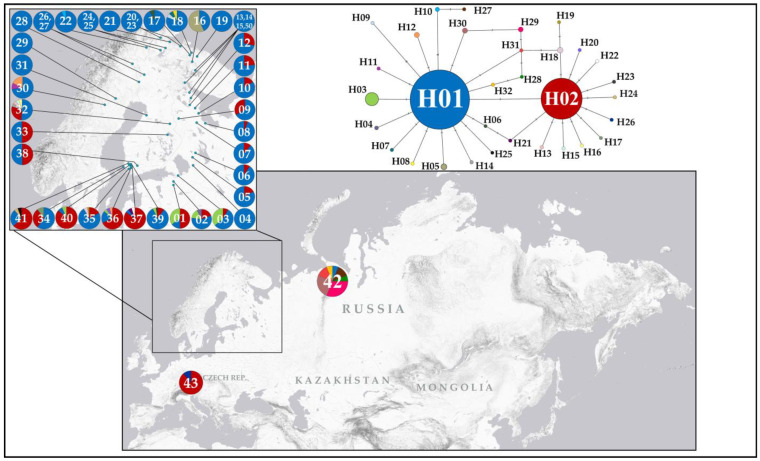
Geographical distribution of *COI* haplotypes and a haplotype network illustrating the relationships among the revealed *COI* haplotypes. Mutations are shown as one-step edges.

Haplotype analysis of the dataset comprising 925 specimens revealed 32 closely related *COI* haplotypes (H01–H32) (Figure 3), with a maximum *p*-distance of 0.92% (±0.35%) (Table 1). Each haplotype differed from its nearest neighbour by no more than two nucleotide substitutions. The haplotype network exhibited a star-like topology, with two major haplotypes (H01 and H02) forming a centre separated by a single nucleotide substitution (A→T at position 205 of the analysed fragment). These two major haplotypes were the most frequent, together accounting for 92.5% of all specimens analysed, and exhibited broad geographical distribution. Haplotype H02 was found in 255 specimens across 22 of the 44 genetically studied localities. Haplotype H01 was recovered in 601 specimens and occurred in all sampled localities, except for the population from the Czech Republic. A third common haplotype, H03, was found in 24 specimens from six populations, all geographically restricted to southern Finland and the Leningrad Oblast (northwestern Russia). Of the 32 haplotypes identified, 28 were private (i.e., found in only one locality), and 21 of these were “singletons” (i.e., found in only a single individual). Fu and Li’s D* test yielded a significantly negative value (−7.42374; *p* < 0.02) (Table 1), indicating an excess of singleton haplotypes, which may suggest recent population expansion or purifying selection. Detailed data on haplotype diversity, their distribution across specimens, and their geographical occurrence are given in Table 1, Table 2 and Table 3.

Haplotype diversity among the populations was generally low. In most localities, only one haplotype (found at 16 sampling sites out of 44 studied) or two haplotypes (found at 14 sites out of 44 studied) were detected (Table 3 and Figure 3). Moderate haplotype diversity was observed in populations located in southern Finland, central Sweden, the Leningrad Oblast and the Russian High North (Murmansk), where 3 to 5 different haplotypes were found in each locality. A relatively high haplotype diversity was recovered only in two geographically distant populations from central Finland (Fi32, six haplotypes) and Vorkuta (Ru42, seven haplotypes). The distribution of haplotypes showed a clear geographical pattern: haplotypes were either widespread across a large area or confined to one or more neighbouring localities.

### 3.2. Sex Ratio in Populations

**Figure 4:**
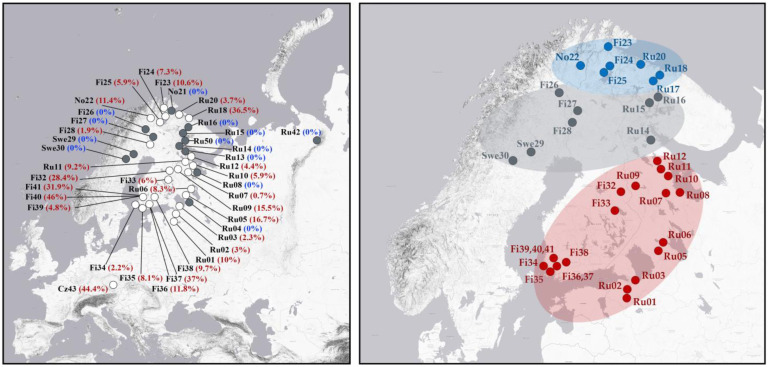
Maps showing male frequency in the studied populations of *C. ledi* (**left**) and the estimated distribution of two bisexual and all-female populations (**right**). Grey and white circles indicate all-female and mixed populations, respectively. Sampling sites where fewer than five specimens were collected are not shown. Male frequencies (in %) are given in brackets. The estimated distribution of ancestral and recently derived bisexual lineages is highlighted in red and blue, respectively; the distribution of all-female populations is highlighted in grey.

A total of 5350 specimens were included in the sex-ratio analysis. Overall, the sex ratio is strongly biased towards females, with 89.3% of all analysed individuals being females. However, the proportion of males varied significantly among populations, ranging from 0% to 46% depending on the geographical region (Figure 4 and Supplementary Table S3), suggesting the presence of different reproductive strategies in this species. Males were found in 31 of the 50 populations examined. The highest male frequencies—approaching parity with females—were observed in populations from the Czech Republic (44.4%), southern Finland (32–46%), and the Russian High North (Murmansk, 36.5%) (Figure 4). Moderate male frequencies (2–16%) were recorded in several populations from northwestern Russia (the Leningrad Oblast, southern Karelia and the northern Murmansk Oblast), as well as in southern and northern Finland and northern Norway. In contrast, all-female populations or populations with single males were found predominantly in Fennoscandia between latitudes 66° and 68° N (Figure 4).

### 3.3. Wolbachia Infection

**Figure 5:**
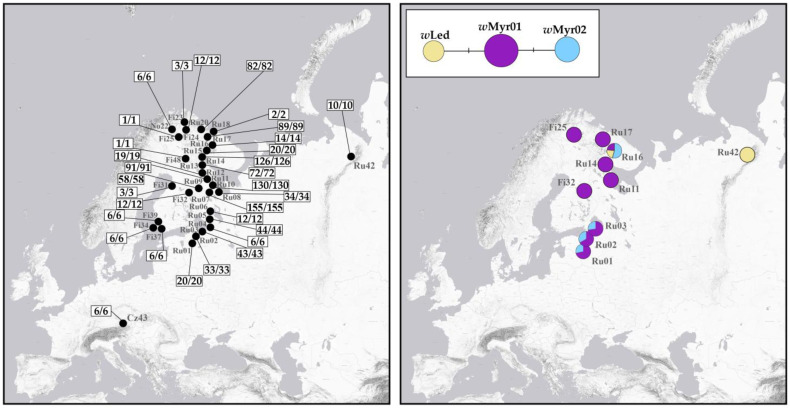
Maps showing *Wolbachia* prevalence (**left**) and the geographical distribution of identified *Wolbachia wsp* alleles (**right**) in *C. ledi*. Numbers represent the ratio of infected specimens (before slash) to analysed specimens (after slash) for each population. Haplotype network illustrating the relationships among the revealed *Wolbachia wsp* alleles is given. Mutations are shown as one-step edges.

A total of 1140 specimens (1059 females, 80 males, and 1 immature individual) from 32 populations across Fennoscandia, northwestern Russia, and the Czech Republic were tested for the presence of *Wolbachia* infection (Figure 5). Of these, 1050 specimens were screened in the present study, 90 specimens were analysed earlier [17]. Overall, all 1140 specimens were positive for *Wolbachia*, indicating a 100% prevalence.

We analysed 44 sequences of a *Wolbachia wsp* gene fragment (549 bp) obtained in this study (31 samples) and in previous studies (13 samples, GenBank accession numbers MZ684119-MZ684131) (Supplementary Table S4). Sequencing revealed three different *wsp* alleles (*w*Myr01, *w*Myr02, and *w*Led) among samples representing ten localities. All three alleles were reported in *Cacopsylla* earlier [17]. The *w*Myr01 and *w*Myr02 alleles differ by a single nucleotide substitution (A→G transition at position 498 of the 549 bp *wsp* gene fragment), while the *w*Led allele differs from the most common *w*Myr01 allele in a G→C transversion at position 499. The geographical distribution of these alleles is shown in Figure 5.

The *w*Myr01 allele is widespread across Fennoscandia and NW Russia, being found in nine of the ten localities analysed. The *w*Myr02 allele was detected in four populations: in one from the Russian High North (Ru16) and in three from the Leningrad Oblast (Ru01, Ru02, and Ru03). In the north (Ru16), the *w*Myr02 allele occurred as a single infection in specimens with haplotype H05, whereas individuals with haplotype H01 always carried three alleles (*w*Myr01, *w*Myr02, and *w*Led), indicating multiple *Wolbachia* infection. This infection pattern was also found in all analysed specimens of *C. borealis* from this locality (our unpublished data). In the south (Ru01-Ru03), the *w*Myr02 allele has never been observed in the parthenogenetic specimens with haplotype H01. It was found exclusively in “bisexual” specimens with haplotypes H02 and H03, which were always combined with the *w*Myr01 allele, suggesting a double infection. The third allele, *w*Led, was found in two geographically remote populations. In the population Ru42 (Vorkuta), it appeared as a single infection, whereas in the population Ru16 (Russian High North), it occurred in each individual along with two other alleles, *w*Myr01 and *w*Myr02.

### 3.4. Cytological Analysis of Ploidy Level and Its Relationship with COI Haplotypes

We determined the ploidy of females in five populations with varying frequencies of males. In a population from the Russian High North (Ru18, male frequency 36.46%), all 30 examined females were diploid, each showing 13 bivalents (12AA + XX). These females carried either the main haplotype H01 (27 individuals), or one of its singleton derivatives: H07, H08, or H09. Additionally, three males from a nearby locality (Ru20) were examined. All of them showed normal meiosis and produced functional sperm, confirming their potential for successful mating with diploid females. In another population from southern Finland (Fi37) with an equally high male frequency (37.02%), 42 out of the 43 analysed females were diploid. They carried the second main haplotype H02 (40 individuals) or one of its singleton satellites, H21 or H22. Interestingly, the only triploid female found in this population (with 39 univalent chromosomes (36A + XXX)) carried the main haplotype H01, which is common among diploid females in Ru20. These findings suggest that both populations are of bisexual origin, although rare parthenogenetic triploid females may occur within such populations, as observed in Fi37. In the population with a moderate male frequency (13.90%) from southern Finland (Fi41b), both triploid and diploid females were present, with diploids predominating.

All diploid females carried the main *COI* haplotype H02 (24 individuals), whereas triploid females carried either the main haplotype H01 (14 individuals) or its singleton derivative H25. In two populations with a relatively low frequency of males—from northern Finland (Fi25, 5.88%) and southern Finland (Fi35, 8.06%)—both diploid and triploid females were observed, with triploids strongly dominating the population structure. However, in the population from northern Finland (Fi25), both diploid and triploid females shared the haplotype H01. In contrast, in southern Finland (Fi35), they carried different main haplotypes: all triploid females (26 individuals) had the haplotype H02, while diploid females carried either H02 (five individuals), or its singleton derivative H19 (see Table 4). The cytological data we obtained demonstrate a strong correlation between the ploidy level of females and the frequency of males in *C. ledi* populations and suggest that the analysed populations from southern (Fi35, Fi41) and northern (Fi25) Finland are of mixed origin and comprise both bisexual and parthenogenetic lineages.

## 4. Discussion

In this study, we employed an integrative approach that combines molecular, cytogenetic and phylogeographic data to study population structure, reproductive strategies, evolutionary history, *Wolbachia* diversity and infection patterns of the widespread psyllid species *Cacopsylla ledi*.

### 4.1. Phylogeographic Patterns Depending on the Mode of Reproduction

The haplotype network analysis of mitochondrial DNA barcodes recovered a star-like topology (Figure 3), known to be characteristic of recent population expansions of the species, likely reflecting the processes of recolonization after the last glacial maximum. A similar genetic structure with two widespread main haplotypes and a number of satellite haplotypes was previously identified in *C. myrtilli* [16]. Notably, most haplotypes detected in *C. ledi* were singletons. Such a network structure (the main haplotype (-es) with numerous singleton satellites) is a frequently observed phenomenon in insects [44,45,46]. The two main haplotypes, H01 and H02, were the most common and widespread, although their distribution was not uniform. The haplotype H01 was found throughout the studied area, except for one population in southern Bohemia (Cz43). In contrast, the haplotype H02 was confined to southern and central Fennoscandia, southern parts of northwestern Russia, and it was also found in a geographically remote locality in the Czech Republic (Červené Blato) (Figure 3). This haplotype reaches a latitude 65–66° N, not spreading further north. Despite extensive sampling in Scandinavia and northwestern Russia, no specimens carrying the H02 haplotype were found north of latitude 65.62° N. In general, *C. ledi* exhibits a low haplotype diversity. Four or more haplotypes were found in only a few localities restricted to narrow zones in the eastern part of central Finland, southern Finland, and the northern Murmansk Oblast (Russia) (Table 3). These haplotypes are mainly found in single individuals, being deviant variants that are not fixed in populations. This pattern suggests a recent and relatively rapid expansion of *C. ledi* in Fennoscandia, most likely associated with post-glacial recolonization. Interestingly, in the closely related species *C. myrtilli*, a much higher haplotype diversity (six to nine haplotypes) has been observed in some parts of Fennoscandia, especially in *Wolbachia*-free populations from southern Norway [16].

Recent studies [8,16] have demonstrated that *C. ledi* has a complex population structure. In southern Fennoscandia, the bisexual lineage has an ancestral origin, as indicated by its unique mitochondrial haplotype, which is not found in the parthenogenetic lineage. In contrast, the bisexual lineage found in northern Fennoscandia probably arose because of recent secondary transition (reversion) from parthenogenesis. Our data confirm this conclusion by shaping the distribution of lineages with different reproductive modes across Fennoscandia and northwestern Russia. We found that all-female populations predominantly occupy a relatively narrow zone of Fennoscandia between latitudes 66–68° N (Figure 4). In addition, males were not detected in populations from northern Norway (No21), southern Karelia (Ru08), and the northern Leningrad Oblast (Ru04). These results may reflect either a mosaic distribution of fully parthenogenetic populations or be the result of an insufficient sampling. The latter seems likely, especially for the Karelian population (Ru08), where only 18 female specimens were collected. It is noteworthy that one of the analysed females from this locality carried the H02 haplotype, typically associated with bisexuals (see explanations below), which suggests the presence of males in this population, at least in small numbers. In two other all-female populations, No21 and Ru04, a much larger number of *C. ledi* specimens were analysed (42 and 50, respectively). Thus, even if males do occur in these localities, their frequency in the population should be very low. Moreover, none of the 41 females sequenced from Ru04 carried the bisexual haplotype H02, indicating that all the analysed specimens are triploids.

In *C. myrtilli*, triploid apomictic females produce rare non-functional males and diploid females at equal frequencies of up to 11% [6,7]. In our study of *C. ledi*, the frequency of males was significantly higher in most populations, where both sexes were present, suggesting possible bisexual reproduction. However, the functionality of these males must be confirmed by cytological analysis of meiosis, which has not yet been done. Cytological and haplotype analyses confirmed the mixed structure of these populations, represented by both bisexual and parthenogenetic lineages occurring syntopically and synchronously. In addition, populations with very high frequencies of males (32–46%) were found in geographically remote regions, including the Russian High North, southern Scandinavia, and Central Europe, suggesting fully bisexual reproduction, although the presence of sporadic parthenogens and rare cases of parthenogenetic reproduction in these populations cannot be completely ruled out. We analysed the ploidy levels of females in five selected populations with different frequencies of males. The results of cytological analysis agree well with the inferred sex ratios and reproductive strategies (parthenogenetic, mixed, or bisexual) for these populations (Supplementary Table S3). Specifically, in populations with the highest proportion of males (almost equal to the proportion of females), the analysed females were almost exclusively diploid, supporting the conclusion that these populations are bisexual. These findings are consistent with the general assertion that a population can be considered bisexual if at least 30% of individuals are males [47]. In populations with the proportion of males less than 30%, both diploid and triploid females were found, indicating a mixed composition of these populations, consisting of both bisexual and parthenogenetic lineages. The ratio of diploid to triploid females strongly correlates with the frequency of males: the lower the frequency of males, the higher the proportion of triploid females in the population.

One of the most intriguing results of our study is that in a vast geographical area covering southern and central Fennoscandia and southern northwestern Russia, parthenogenetic and bisexual lineages co-existing within the same populations can be distinguished by mitochondrial DNA barcodes alone. Diploid females and functional males in this region carry the main haplotype H02 or its satellite haplotypes, whereas triploid females invariably carry the main haplotype H01 or its derivatives. This pattern persists in populations with low frequencies of males and diploid females, suggesting that mitochondrial DNA barcodes provide a clear genetic distinction between parthenogenetic and bisexual individuals. It remains unclear whether functional males can non-specifically mate with both abundant triploid (parthenogenetic) and rare diploid (“bisexual”) females, or whether they are able to distinguish between these two types of females. Our study also highlighted rare cases where parthenogenetic *C. ledi* females produce rare functional males. For example, in the Levonsuo population (Fi39) in southern Finland, which reproduces predominantly by parthenogenesis, a male carrying the same haplotype as triploid females was found, indicating a functional reversion from triploidy.

A completely different pattern was observed in northern Fennoscandia, where either mixed or completely bisexual populations were found. In contrast to southern Fennoscandia, all males and diploid females in the northern populations shared the same haplotype as triploid females. Bisexual reproduction is considered ancestral to parthenogenesis [48]. Thus, the pattern observed in northern Scandinavia and the Russian High North appears to represent a secondary reversion from parthenogenesis to bisexual reproduction, which likely occurred very recently in the evolutionary history of *C. ledi*, since bisexual individuals have not yet accumulated nucleotide differences in their DNA barcodes.

According to the concept of geographic parthenogenesis [49,50,51], parthenogenetic species or populations tolerate harsh climatic conditions better, often colonising higher latitudes and altitudes than their bisexual relatives. The phylogeographic pattern revealed in our study suggests that the current distribution of parthenogenetic and bisexual lineages of *C. ledi* is closely linked to the processes of recent post-glacial recolonization. After the retreat of the ice sheets, the bisexual lineage reached the White Sea, central Karelia and southern Finland, while the parthenogenetic lineage spread further north, colonising areas with more severe climatic conditions. Subsequently, the parthenogenetic lineage gave rise to secondary bisexual populations, most likely when climatic conditions became more favourable for the survival and persistence of bisexual individuals. This bisexual lineage is geographically well isolated (by ca. 400 km) from the ancestral bisexual populations (Figure 4) and currently descends from triploid ancestors. This geographic isolation may promote further divergence between the two bisexual lineages, potentially leading to the formation of a new species.

### 4.2. Wolbachia Infection

*Wolbachia* is one of the most common endosymbiotic bacteria in terrestrial arthropods, known for its diverse impacts on host species. *Wolbachia* can affect the reproduction and physiology of the host, causing feminization, altering the sex ratios by male-killing, prompting parthenogenesis, or causing cytoplasmic incompatibility. In the latter case, infected females can successfully mate only with uninfected males or males infected with the same *Wolbachia* strain [52,53,54]. In addition, *Wolbachia* has been shown to drive diversification and speciation in its hosts [55,56]. It can also induce mitochondrial selective sweeps due to genetic “hitchhiking”, leading to mito-nuclear discordance and potential misinterpretations of mtDNA-based phylogenies [28,57,58].

To study the impact of *Wolbachia* on *C. ledi* and to assess whether the endosymbiont influences the parthenogenetic and bisexual reproductive patterns observed in this species, we performed PCR screening for three *Wolbachia* genes based on a large-scale and geographically extensive sampling. Unlike the closely related *Cacopsylla* species studied earlier [17], which showed a complex pattern of *Wolbachia* prevalence with varying infection levels in populations, including both infected and uninfected ones, all 1140 *C. ledi* specimens from 32 populations were infected with *Wolbachia*. Analysis of the *Wolbachia wsp* gene fragment revealed three closely related alleles in *C. ledi*, none of which was species-specific, being shared with at least one other *Cacopsylla* species. The most common allele, *w*Myr01, was detected in most *C. ledi* specimens, as well as in several other *Cacopsylla* species, including *C. myrtilli*, *C. borealis*, *C. lapponica*, and *C. fraudatrix*. The *w*Myr02 allele was found in *C. ledi* specimens from two regions: northern Murmansk and the Leningrad Oblast. It has also been detected in *C. borealis* (unpublished data) and *C. myrtilli* [17]. In the latter species, the *w*Myr02 was found exclusively in a geographically remote population from Magadan (the Russian Far East), which indicates the wide distribution of this allele across the Palaearctic. In *C. ledi*, the *w*Myr02 strain was present either as a single infection (in specimens with the H05 haplotype, unique to the population Ru16), or combined with the *w*Myr01 (in Leningrad Oblast) and with the *w*Myr01 and *w*Led alleles (in specimens with the H01 haplotype from the population Ru16), indicating a co-infection with two or even three *Wolbachia* alleles. It is noteworthy that in the Leningrad Oblast, the *w*Myr02 allele was characteristic exclusively of “bisexual” specimens (haplotypes H02 and H03) and was never detected in the parthenogenetic lineage (haplotype H01). The third allele, *w*Led, was detected only in a geographically remote population from Vorkuta (Ru42) and in one population from the Russian High North (Ru16). This allele was also identified in specimens of the closely related sympatric species *C. borealis* collected from these localities (unpublished data). In Ru42, *w*Led occurred as a single infection, while in Ru16, it was always found together with two other alleles, *w*Myr01 and *w*Myr02, suggesting multiple *Wolbachia* infection. Given that *C. borealis* utilises the same host plant (*Ledum palustre*) as *C. ledi*, the presence of identical *Wolbachia* alleles in both species suggests potential interspecific horizontal transmission through a common food substrate.

Our study revealed no differences in *Wolbachia* infection rates between males and females, between specimens with different ploidy levels (diploids and triploids), and between parthenogenetic (“female-only”), genuine bisexual, and mixed populations (where two reproductively isolated lineages, reproducing parthenogenetically and bisexually, co-exist in the same population). A well-known effect of *Wolbachia* is a decrease in mtDNA polymorphism in host species [22,59,60]. However, we did not detect such an effect. Our data suggest that haplotype diversity in some *C. ledi* populations varies significantly irrespective of *Wolbachia* infection. We also found that *Wolbachia* alleles detected in *C. ledi* are not limited to a specific *COI* haplotype (-es), as has been shown in other insects such as Lepidoptera [33,61].

## 5. Conclusions

1. The plant louse *C. ledi* exhibits two different reproductive strategies throughout its distribution range: parthenogenetic and bisexual. Parthenogens are geographically widespread and have been found in all studied populations, with the exception of an isolated Central European population in the Southern Bohemia. Bisexuals occupy two geographically separated zones and appear to have emerged at least twice in the evolutionary history of *C. ledi*. Bisexuals found in central Europe and southern Fennoscandia are of ancestral origin, while those inhabiting northern Fennoscandia have emerged recently as a reversion from a parthenogenetic lineage.
2. Haplotype analysis of DNA barcodes revealed a star-like structure with two dominant, geographically widespread major haplotypes and a number of satellite, mainly private haplotypes. This structure indicates a relatively rapid species expansion of *C. ledi*, most likely associated with post-glacial recolonization processes.
3. The ancestral bisexual lineage is characterised by specific *COI* haplotypes that were not found in parthenogens, and it can be easily distinguished using DNA barcoding alone.
4. Haplotype diversity, reproductive strategies, and shifts in modes of reproduction in *C. ledi* are not associated with *Wolbachia* infection. They are likely influenced by other factors, most probably environmental or biogeographical, which warrants further study. Nonetheless, the presence of *Wolbachia* infection in all analysed *C. ledi* specimens suggests that the endosymbiont plays an important role in the biology and evolution of the species, potentially contributing to the observed biogeographic patterns. No other *Cacopsylla* species previously screened for *Wolbachia* had such a total infection.

## Figures and Tables

**Table 1:** Summary of the mitochondrial haplotype diversity. The number of sequenced individuals (*N*), number of revealed haplotypes (*H*), number of polymorphic sites (*S*), nucleotide (*π*) and haplotype (*h*) diversity are given. Tajima’s D, Fu and Li’s D, Fu and Li’s F, Fu’s Fs, the max. *p*-distance values with standard deviations (in parentheses) are shown.

*N*	*H*	*S*	*π*	*h*	Tajima’s D	Fu and Li’s D*	Fu and Li’s F	Fu’s Fs	Max. P-Dist.
925	32	29	0.00092	0.521	−2.12636	−7.42374 *p* < 0.02	−6.17885 *p* < 0.02	−44.034	0.92% (±0.35%)

**Table 2:** Composition of revealed *COI* haplotypes (*n*—number of individuals sharing a given haplotype, *Sn*—number of sampling sites, in which the haplotype was found).

Haplotype	*n*	*Sn*	Sampling Site
H01	601	43	Ru01–Ru020, Ru42, Ru50, No21, No22, Swe29, Swe30, Fi23–Fi41
H02	255	22	Ru01–Ru03, Ru05–Ru12, Fi32–Fi41, Cz43
H03	24	6	Ru01–Ru03, Fi036, Fi039, Fi040
H04	1	1	Ru02
H05	4	1	Ru16
H06	1	1	Ru17
H07	1	1	Ru18
H08	1	1	Ru18
H09	1	1	Ru18
H10	2	1	No22
H11	1	1	Swe30
H12	3	2	Swe30, Fi36
H13	1	1	Fi32
H14	1	1	Fi32
H15	1	1	Fi32
H16	1	1	Fi32
H17	1	1	Fi34
H18	2	1	Fi35
H19	1	1	Fi35
H20	1	1	Fi36
H21	1	1	Fi37
H22	1	1	Fi37
H23	1	1	Fi39
H24	1	1	Fi41
H25	1	1	Fi41
H26	1	1	Cz43
H27	2	1	Ru42
H28	1	1	Ru42
H29	5	1	Ru42
H30	4	1	Ru42
H31	2	1	Ru42
H32	1	1	Ru42

**Table 3:** Geographical distribution of revealed *COI* haplotypes. *H*—number of haplotypes found in a sampling site; *Sn*—number of sampling sites, in which a certain number of haplotypes (*H*) was found.

*H*	*Sn*	Sampling Site
7	1	Ru42
6	1	Fi32
5	1	Fi36
4	6	Fi35, Fi37, Fi39, Fi41, Ru02, Ru018
3	5	Fi30, Fi34, Fi40, Ru01, Ru03,
2	14	Ru05–Ru12, Ru16, Ru17, No22, Fi33, Fi38, Cz43
1	16	Ru04, Ru13–Ru15, Ru19, Ru20, Ru50, Fi23, No21, Fi24-Fi29, Fi31

**Table 4:** Proportions of triploid (3n) and diploid (2n) females, and their correlation with revealed COI haplotypes in five examined populations. The number of individuals sharing a given haplotype is given in brackets.

Population	Male Frequency	Females 3n/2n	*COI* Haplotypes	Reference
Ru18	36.46%	0/30	-/H01 (27), H07 (1), H08 (1), H09 (1)	Present study
Fi37	37.02%	1/42	H01 (1)/H02 (40), H21 (1), H22 (1)	[9]
Fi41	13.90%	15/24	H01 (14), H25 (1)/H02 (24)	[9]
Fi25	5.88%	36/5	H01 (36)/H01 (5)	[9]
Fi35	8.06%	64/7	H01 (26)/H02 (5), H19 (1)	[8]

## Data Availability

The original contributions presented in this study are included in the article. Further inquiries can be directed to the corresponding authors.

## Supplementary Materials

The following supporting information can be downloaded at: https://www.mdpi.com/article/10.3390/insects16121268/s1, Table S1: List of collected material; Table S2: List of sampling localities and sequenced individuals (males and females) of *Cacopsylla ledi* sequenced from each locality. Asterisk (*) indicates an immature individual; Table S3: Sex ratio (number of males and females) and male frequency with 95% confidence intervals (in brackets) in studied populations of *C. ledi*; Table S4: List of specimens sequenced for Wolbachia wsp gene.
