# Supplementary material for: Genetic Differentiation of Bisexual and Parthenogenetic Populations of Plant Louse Cacopsylla ledi (Hemiptera, Psylloidea)
Source: Insects. 2025 Dec 13;16(12):1268. doi: 10.3390/insects16121268 (PMC12733736; doi:10.3390/insects16121268)
Supplement: Supplementary file 1 [file insects-16-01268-s001.zip › Supplementary02.pdf]

Table S2. List of sampling localities and sequenced individuals (males and females) of *Cacopsylla ledi* sequenced from each locality. Asterisk (*) indicates an immature individual.

Locality	Coordinates	Specimens sequenced
RU_01 RUSSIA, LO, Mshinskaya	59.038101, 29.934211	18♀ + 2♂
RU_02 RUSSIA, LO, Rozhdestveno	59.296943, 29.928911	24♀ + 1♂
RU_03 RUSSIA, LO, Razmetelevo	59.904348, 30.794841	24♀ + 1♂
RU_04 RUSSIA, LO, Lodeynoye Pole	60.790191, 33.406198	41♀
RU_05 RUSSIA, KA, Petrozavodsk	61.460329, 33.313781	5♀ + 1♂
RU_06 RUSSIA, KA, Petrozavodsk	61.789727, 33.846539	10♀ + 1♂
RU_07 RUSSIA, KA, Pushnoy	64.163324, 34.119290	8♀ + 1♂
RU_08 RUSSIA, KA, Kolezhma	64.246033, 35.813505	18♀
RU_09a RUSSIA, KA, Kostomuksha	64.422681, 31.314972	2♀+ 2♂
RU_09b RUSSIA, KA, Kostomuksha	64.505856, 31.112329	2♀
RU_10 RUSSIA, KA, Kem 1	64.928500, 34.456145	11♀ + 2♂
RU_11 RUSSIA, KA, Kem 2	65.260076, 33.747531	15♀+2♂
RU_12 RUSSIA, KA, Loukhi 1	65.624046, 33.178799	5♀+2♂
RU_13 RUSSIA, KA, Loukhi 2	66.034695, 32.977941	19♀
RU_14 RUSSIA, KA, Tedino	66.467200, 32.770413	24♀
RU_15 RUSSIA, MU, Monchegorsk	67.988485, 32.903390	10♀
RU_16 RUSSIA, MU, Taibola	68.508636, 33.325378	7♀
RU_17 RUSSIA, MU, Murmansk 1	68.983736, 32.990403	6♀ + 9♂
RU_18 RUSSIA, MU, Mokhnatkina Pakhta	69.050851, 33.161461	30♀ + 24♂
RU_19 RUSSIA, MU, Murmansk 2	68.887435, 34.452951	1♀ + 1(*)
RU_20 RUSSIA, MU, Pechenga	69.521094, 31.273108	3♀ + 1♂
RU_42 RUSSIA, KO, Vorkuta	67.459444, 63.983611	16♀
RU_50 RUSSIA, MU, Kandalaksha	67.157293, 32.150432	12♀
NO_21 NORWAY, Neiden	69.732586, 29.296901	11♀
NO_22 NORWAY, Karasjok	69.443289, 25.193346	20♀ + 12♂
NO_47 NORWAY, Sjoa	61.687500, 09.240833	0
SW_29 SWEDEN, Kåbdalis	66.027386, 19.908879	9♀
SW_30 SWEDEN, Sorsele	65.571807, 18.045919	11♀
FI_23 FINLAND, Utsjoki	70.033325, 27.969737	9♀ + 7♂
FI_24 FINLAND, Nitsijärvi	69.302000, 28.107000	5♀ + 2♂
FI_25 FINLAND, Sevettijärvi	69.216111, 27.870556	42♀ + 6♂
FI_26 FINLAND, Enontekiö	68.324500, 22.993397	11♀
FI_27 FINLAND, Kittilä	67.628000, 24.933000	10♀
FI_28 FINLAND, Kolari	67.209167, 23.906389	10♀ + 1♂
FI_31 FINLAND, Liminka	64.731389, 25.384444	2♀
FI_32 FINLAND, Kuhmo	64.194600, 29.293200	41♀ + 27♂
FI_33 FINLAND, Nurmes	63.335394, 28.828826	6♀ + 6♂
FI_34 FINLAND, Kustavi	60.655556, 21.303333	6♀ + 4♂
FI_35 FINLAND, Turku	60.498889, 22.265278	63♀ + 4♂
FI_36 FINLAND, Marttila	60.544444, 22.444722	6♀ + 16♂
FI_37 FINLAND, Lammenrahka	60.676944, 22.425556	43♀ + 27♂
FI_38 FINLAND, Tammela	60.738889, 23.585778	6♀ + 6♂
FI_39 FINLAND, Levonsuo	60.886111, 22.444722	36♀ + 5♂
FI_40 FINLAND, Valastensuo	60.856000, 22.273000	17♀ + 11♂
FI_41a FINLAND, Isosuo	60.901389, 22.161944	7♂
FI_41b FINLAND, Laustinrahka	60.889722, 22.146667	39♀ + 11♂
FI_44 FINLAND, Heitala	69.851667, 27.009444	0
FI_45 FINLAND, Nurmijärvi	63.543000, 29.963500	0

FI_46 FINLAND, Tokrajärvi	62.754400, 30.577300	0
FI_48 FINLAND, Tuntsantie	67.303056, 29.271667	0
FI_49 FINLAND, Pitkävuono	68.982222, 26.955000	0
CZ_43 CZECH REPUBLIC, Červené Blato	48.858611, 14.803889	7♀ + 2♂
