# Supplementary material for: Genetic Differentiation of Bisexual and Parthenogenetic Populations of Plant Louse Cacopsylla ledi (Hemiptera, Psylloidea)
Source: Insects. 2025 Dec 13;16(12):1268. doi: 10.3390/insects16121268 (PMC12733736; doi:10.3390/insects16121268)
Supplement: Supplementary file 1 [file insects-16-01268-s001.zip › Supplementary03.pdf]

Table S3. Sex ratio (number of males and females) and male frequency with 95% confidence intervals (in brackets) in studied populations of *C. ledi*.

Locality	Coordinates	♀	♂	% of ♂	Reference
RU01	59.038101, 29.934211	18	2	10.00% (±5.08%)	Present study
RU02	59.296943, 29.928911	32	1	3.03% (±1.26%)	Present study
RU03	59.904348, 30.794841	42	1	2.33% (±0.85%)	Present study
RU04	60.790191, 33.406198	50	0	0.00% (n/a)	Present study
RU05	61.460329, 33.313781	5	1	16.66% (±16.40%)	Present study
RU06	61.789727, 33.846539	11	1	8.33% (±5.42%)	Present study
RU07	64.163324, 34.119290	154	1	0.65% (±0.24%)	Present study
RU08	64.246033, 35.813505	18	0	0.00% (n/a)	Present study
RU09a, RU09b	64.422681, 31.314972, 64.505856, 31.112329	49	9	15.52% (±4.86%)	Present study
RU10	64.928500, 34.456145	32	2	5.88% (±2.25%)	Present study
RU11	65.260076, 33.747531	118	12	9.23% (±1.82%)	Present study
RU12	65.624046, 33.178799	87	4	4.40% (±1.04%)	Present study
RU13	66.034695, 32.977941	19	0	0.00% (n/a)	Present study
RU14	66.467200, 32.770413	72	0	0.00% (n/a)	Present study
RU15	67.988485, 32.903390	20	0	0.00% (n/a)	Present study
RU16	68.508636, 33.325378	14	0	0.00% (n/a)	Present study
RU17	68.983736, 32.990403	80	9	10.11% (±2.43%)	Present study
RU18	69.050851, 33.161461	115	66	36.46% (±8.43%)	[9]
RU19	68.887435, 34.452951	1	0	0.00% (n/a)	Present study
RU20	69.521094, 31.273108	79	3	3.66% (±0.92%)	Present study
RU42	67.459444, 63.983611	16	0	0.00% (n/a)	[9]
RU50	67.157293, 32.150432	126	0	0.00% (n/a)	Present study
NO21	69.732586, 29.296901	42	0	0.00% (n/a)	[9]
NO22	69.443289, 25.193346	218	28	11.38% (±1.67%)	[9]
NO47	61.687500, 09.240833	3	0	0.00% (n/a)	[9]
SW29	66.027386, 19.908879	34	0	0.00% (n/a)	Present study
SW30	65.571807, 18.045919	11	0	0.00% (n/a)	Present study
FI23	70.033325, 27.969737	202	24	10.62% (±1.61%)	Present study
FI24	69.302000, 28.107000	41	3	7.32% (±2.31%)	[9]
FI25	69.216111, 27.870556	160	10	5.88% (±1.02%)	[9]
FI26	68.324500, 22.993397	11	0	0.00% (n/a)	Present study
FI27	67.628000, 24.933000	184	0	0.00% (n/a)	[9]
FI28	67.209167, 23.906389	51	1	1.92% (±0.65%)	[9]
FI31	64.731389, 25.384444	2	0	0.00% (n/a)	Present study
FI32	64.194600, 29.293200	161	64	28.44% (±5.26%)	[9]
FI33	63.335394, 28.828826	94	6	6.00% (±1.34%)	[9]
FI34	60.655556, 21.303333	313	7	2.19% (±0.30%)	[9]
FI35	60.498889, 22.265278	1141	100	8.06% (±0.51%)	[9]
FI36	60.544444, 22.444722	202	27	11.79% (±3.44%)	[9]
FI37	60.676944, 22.425556	182	107	37.02% (±6.84%)	[9]
FI38	60.738889, 23.585778	56	6	9.68% (±2.79%)	[9]
FI39	60.886111, 22.444722	102	5	4.67% (±1.02%)	[9]
FI40	60.856000, 22.273000	34	29	46.03% (±21.19%)	[9]
FI41a, FI41b	60.889722, 22.146667, 60.901389, 22.161944	192	31	13.90% (±2.18%)	[9]
FI44	69.851667, 27.009444	28	2	6.66% (±2.73%)	[9]
FI45	63.543000, 29.963500	77	1	1.30% (±0.38%)	[9]
FI46	62.754400, 30.577300	68	0	0.00% (n/a)	[9]

FI48	67.303056, 29.271667	1	0	0.00% (n/a)	Present study
FI49	68.982222, 26.955000	1	0	0.00% (n/a)	Present study
CZ43	48.858611, 14.803889	10	8	44.44% (±37.18%)	[9]
