# Supplementary material for: Genetic Differentiation of Bisexual and Parthenogenetic Populations of Plant Louse Cacopsylla ledi (Hemiptera, Psylloidea)
Source: Insects. 2025 Dec 13;16(12):1268. doi: 10.3390/insects16121268 (PMC12733736; doi:10.3390/insects16121268)
Supplement: Supplementary file 1 [file insects-16-01268-s001.zip › Supplementary04.pdf]

Table S4. List of specimens sequenced for *Wolbachia wsp* gene.

Sample ID	GB Accession No.	Sex	Allele	COI haplotype	Locality	Reference
VORKF2	MZ684129	♀	*w*Led	H28	Ru42 (Russia, Vorkuta)	Shapoval et al. 2021
VORKF3	MZ684130	♀	*w*Led	H29	Ru42 (Russia, Vorkuta)	Shapoval et al. 2021
VORKF6	MZ684131	♀	*w*Led	H29	Ru42 (Russia, Vorkuta)	Shapoval et al. 2021
19EDKAB089	MZ684119	♀	*w*Myr01	H01	FI25 (Finland, Sevettijärvi)	Shapoval et al. 2021
1D34KAA027	MZ684120	♀	*w*Myr01	H01	FI25 (Finland, Sevettijärvi)	Shapoval et al. 2021
1D34KAA030	MZ684121	♀	*w*Myr01	H01	FI25 (Finland, Sevettijärvi)	Shapoval et al. 2021
1D34KAA003	MZ684122	♀	*w*Myr01	H01	FI25 (Finland, Sevettijärvi)	Shapoval et al. 2021
1AB2KAA066	MZ684123	♂	*w*Myr01	H01	FI25 (Finland, Sevettijärvi)	Shapoval et al. 2021
1AB2KAA068	MZ684124	♂	*w*Myr01	H01	FI25 (Finland, Sevettijärvi)	Shapoval et al. 2021
1D7CKAC001	MZ684125	♀	*w*Myr01	H02	FI32 (Finland, Kuhmo)	Shapoval et al. 2021
1D7CKAC002	MZ684126	♀	*w*Myr01	H02	FI32 (Finland, Kuhmo)	Shapoval et al. 2021
1E16KAA094	MZ684127	♂	*w*Myr01	H15	FI32 (Finland, Kuhmo)	Shapoval et al. 2021
1E16KAA098	MZ684128	♂	*w*Myr01	H02	FI32 (Finland, Kuhmo)	Shapoval et al. 2021
C03M1	PX647420	♂	*w*Myr01	H02	RU11 (Russia, Kem 2)	Present study
C03M2	PX647421	♂	*w*Myr01	H02	RU11 (Russia, Kem 2)	Present study
C03F12	PX647422	♀	*w*Myr01	H02	RU11 (Russia, Kem 2)	Present study
C06F10	PX647423	♀	*w*Myr01	H01	RU14 (Russia, Tedino)	Present study
C06F11	PX647424	♀	*w*Myr01	H01	RU14 (Russia, Tedino)	Present study
C13M4	PX647425	♂	*w*Myr01	H01	RU17 (Russia, Murmansk 1)	Present study
C13M5	PX647426	♂	*w*Myr01	H06	RU17 (Russia, Murmansk 1)	Present study
C13F3	PX647427	♀	*w*Myr01	H01	RU17 (Russia, Murmansk 1)	Present study
C13F4	PX647428	♀	*w*Myr01	H01	RU17 (Russia, Murmansk 1)	Present study
C31M1	PX647429	♂	*w*Myr01	H02	RU03 (Russia, Razmetelevo)	Present study
C31F2	PX647430	♀	*w*Myr01	H03	RU03 (Russia, Razmetelevo)	Present study
C31F3	PX647431	♀	*w*Myr01	H01	RU03 (Russia, Razmetelevo)	Present study
C31F5	PX647432	♀	*w*Myr01	H03	RU03 (Russia, Razmetelevo)	Present study
C31F7	PX647433<br>PX647447	♀	*w*Myr01 +<br>*w*Myr02	H02	RU03 (Russia, Razmetelevo)	Present study
C31F17	PX647434	♀	*w*Myr01	H01	RU03 (Russia, Razmetelevo)	Present study
C31F19	PX647435<br>PX647448	♀	*w*Myr01 +<br>*w*Myr02	H03	RU03 (Russia, Razmetelevo)	Present study
C31F20	PX647436<br>PX647449	♀	*w*Myr01 +<br>*w*Myr02	H03	RU03 (Russia, Razmetelevo)	Present study
C32F6	PX647437<br>PX647450	♀	*w*Myr01 +<br>*w*Myr02	H03	RU02 (Russia, Rozhdestveno)	Present study
C32F14	PX647438	♀	*w*Myr01	H03	RU02 (Russia, Rozhdestveno)	Present study
C33M2	PX647439	♂	*w*Myr01	H02	RU01 (Russia, Mshinskaya)	Present study
C33F5	PX647440<br>PX647451	♀	*w*Myr01 +<br>*w*Myr02	H03	RU01 (Russia, Mshinskaya)	Present study
C33F10	PX647441	♀	*w*Myr01	H01	RU01 (Russia, Mshinskaya)	Present study
C33F11	PX647442	♀	*w*Myr01	H01	RU01 (Russia, Mshinskaya)	Present study
C33F17	PX647443<br>PX647452	♀	*w*Myr01 +<br>*w*Myr02	H03	RU01 (Russia, Mshinskaya)	Present study
C12F1	PX647444<br>PX647453<br>PX647460	♀	*w*Myr01 +<br>*w*Myr02+<br>*w*Led	H01	RU16 (Russia, Taibola)	Present study
C12F2	PX647454	♀	*w*Myr02	H05	RU16 (Russia, Taibola)	Present study
C12F3	PX647455	♀	*w*Myr02	H05	RU16 (Russia, Taibola)	Present study
C12F4	PX647445<br>PX647456<br>PX647461	♀	*w*Myr01 +<br>*w*Myr02+<br>*w*Led	H01	RU16 (Russia, Taibola)	Present study
C12F5	PX647446<br>PX647457<br>PX647462	♀	*w*Myr01 +<br>*w*Myr02+<br>*w*Led	H01	RU16 (Russia, Taibola)	Present study
C12F7	PX647458	♀	*w*Myr02	H05	RU16 (Russia, Taibola)	Present study
C12F8	PX647459	♀	*w*Myr02	H05	RU16 (Russia, Taibola)	Present study
